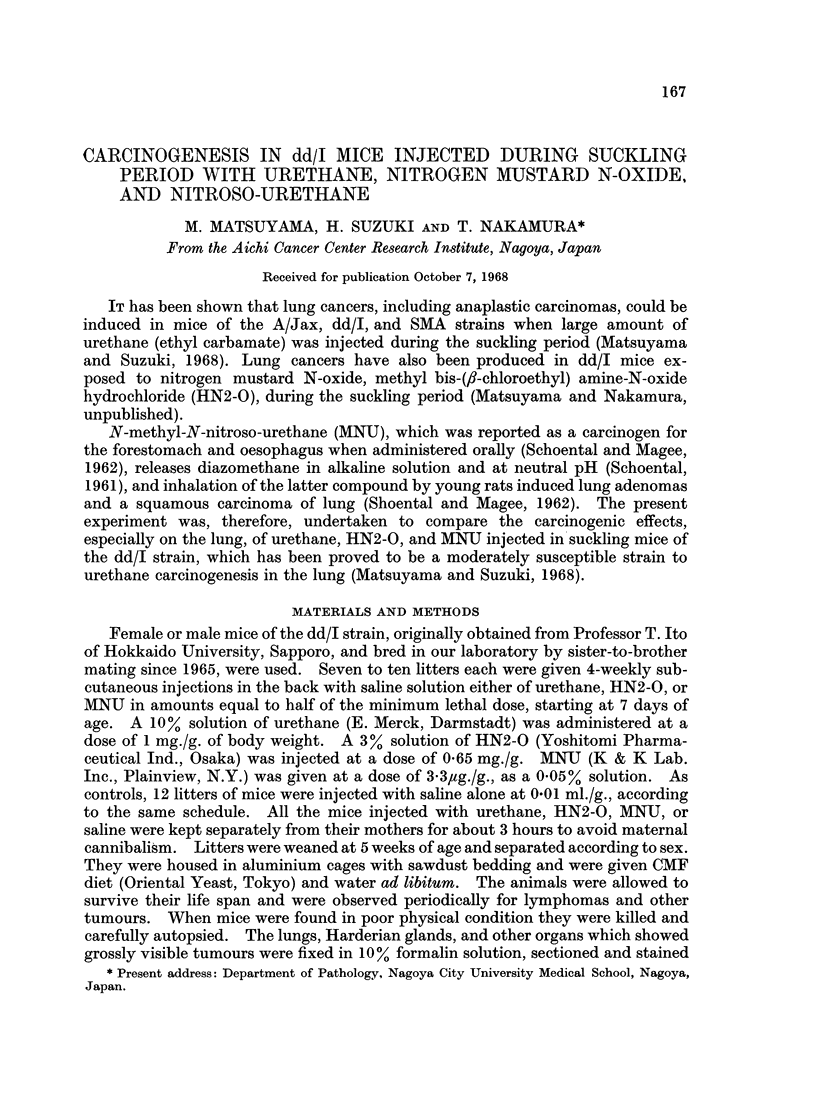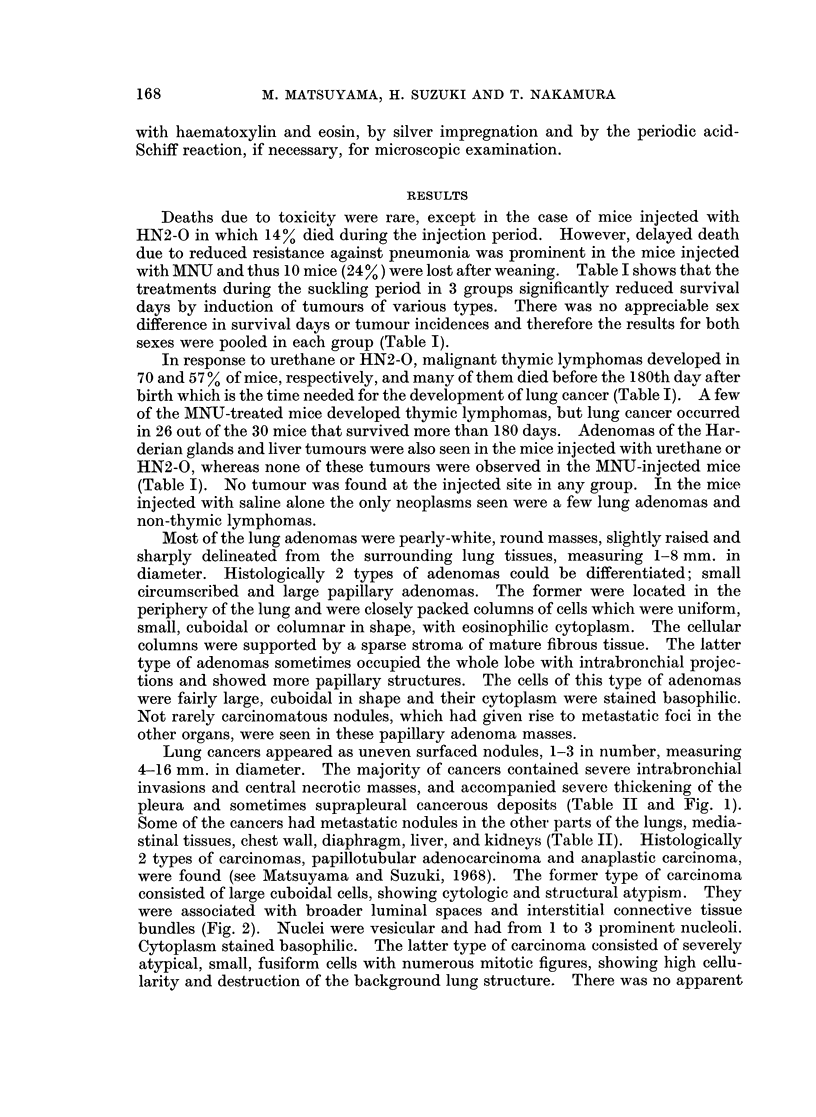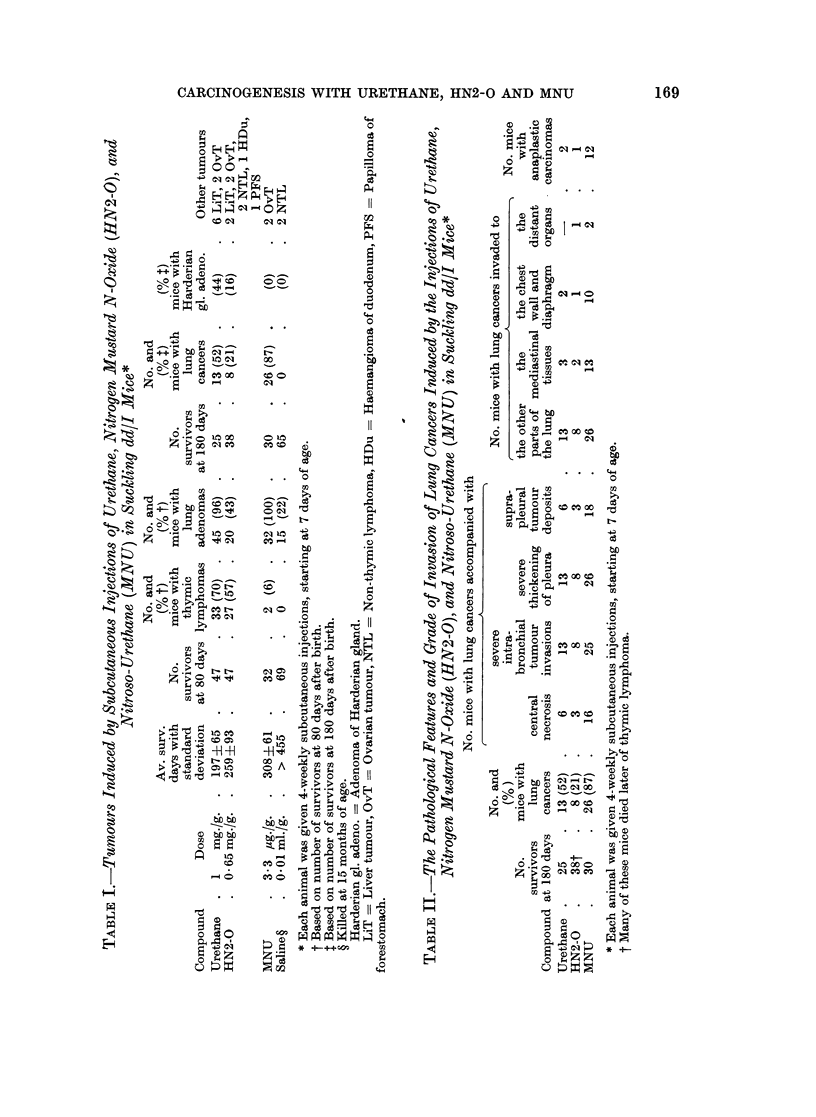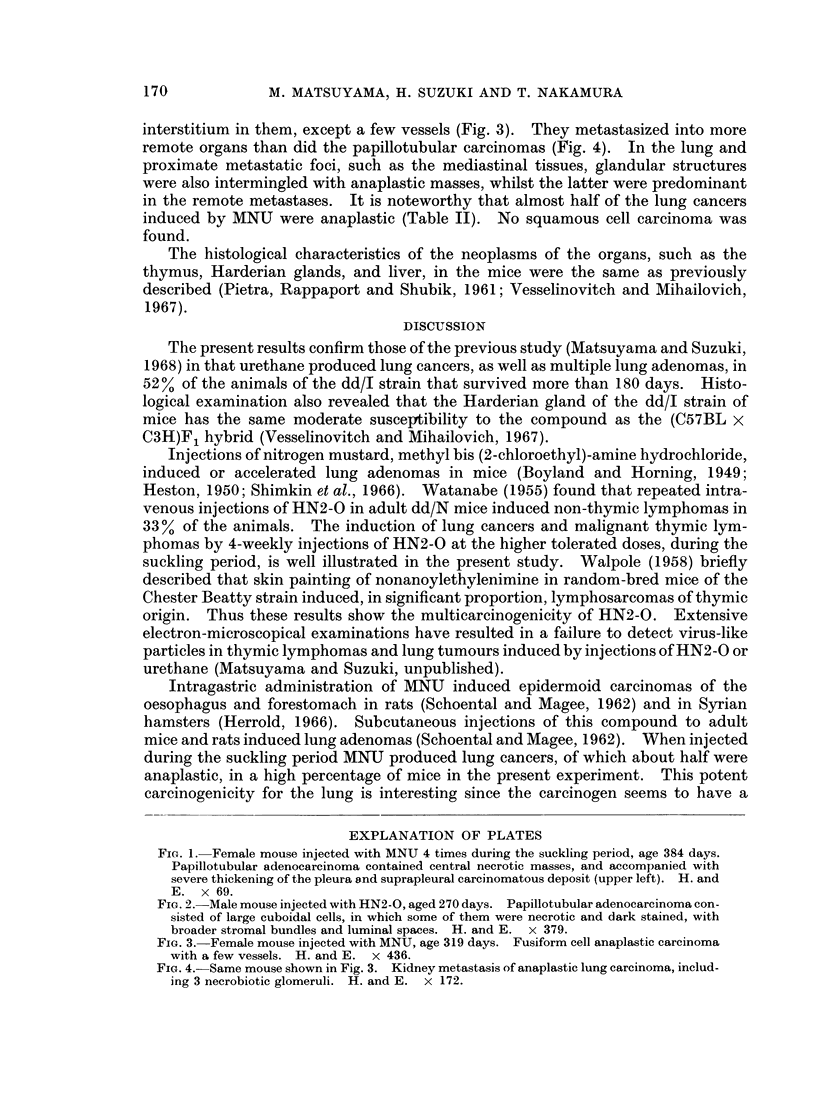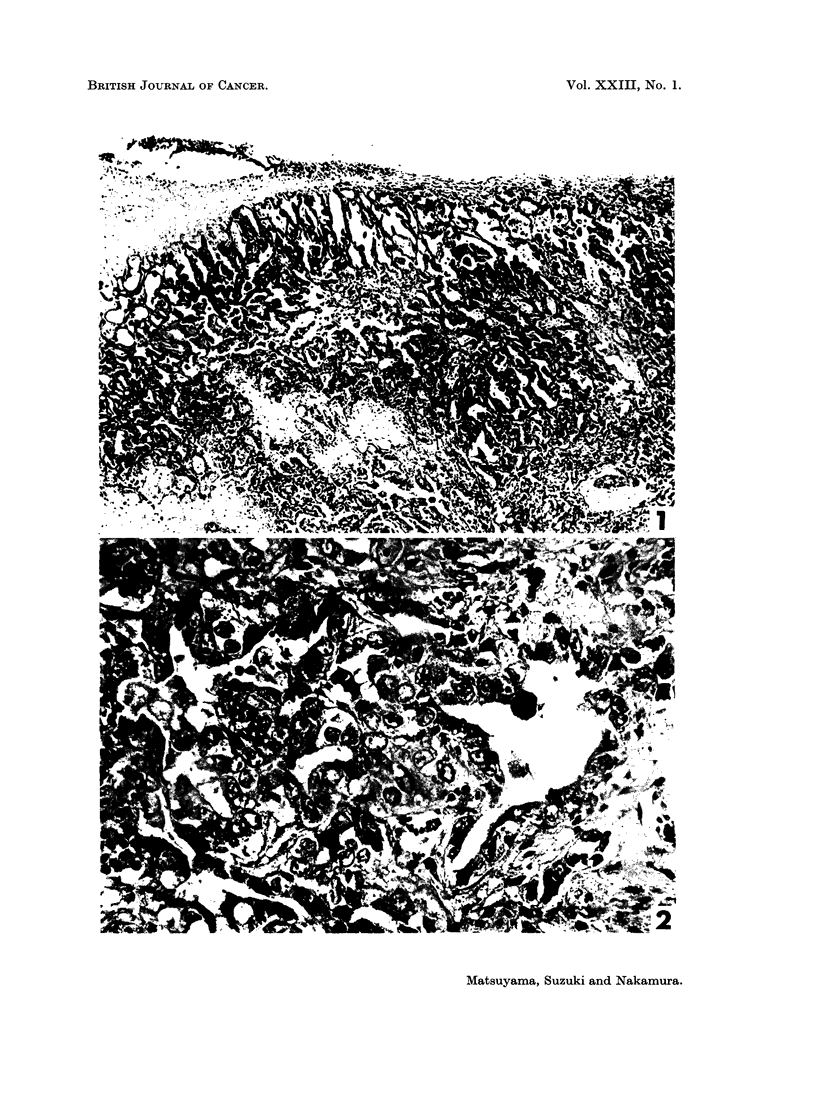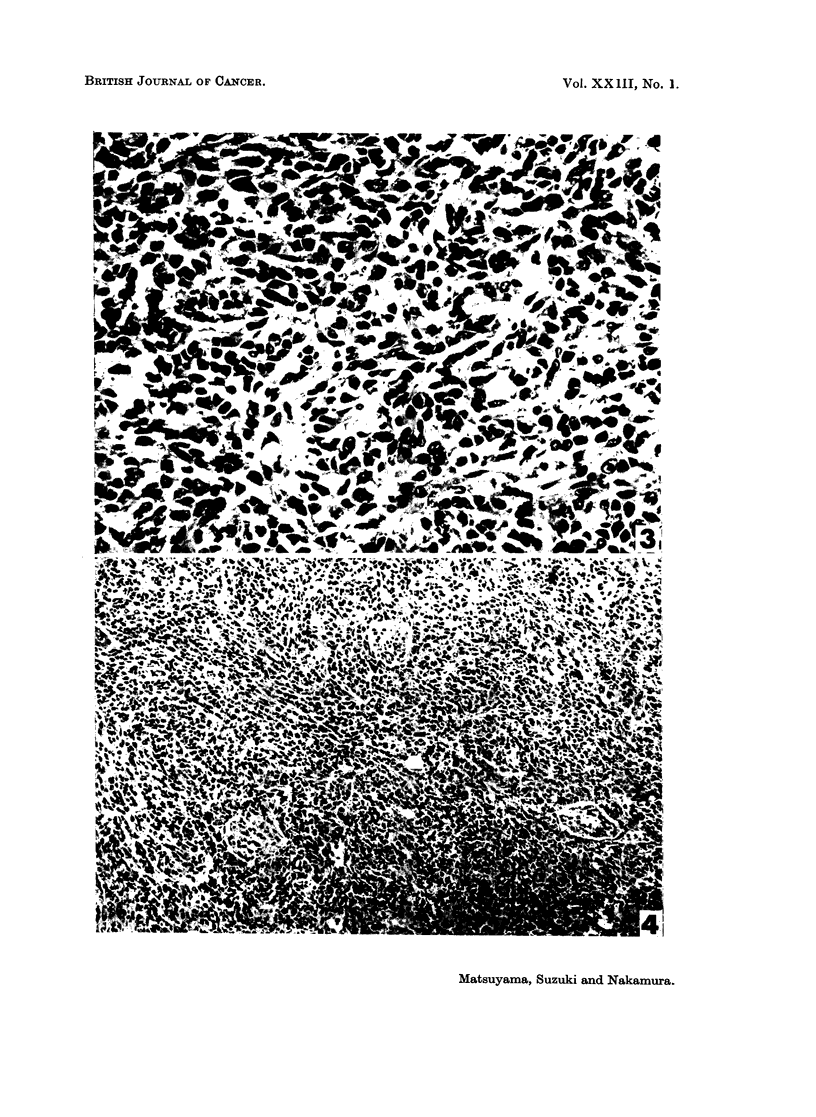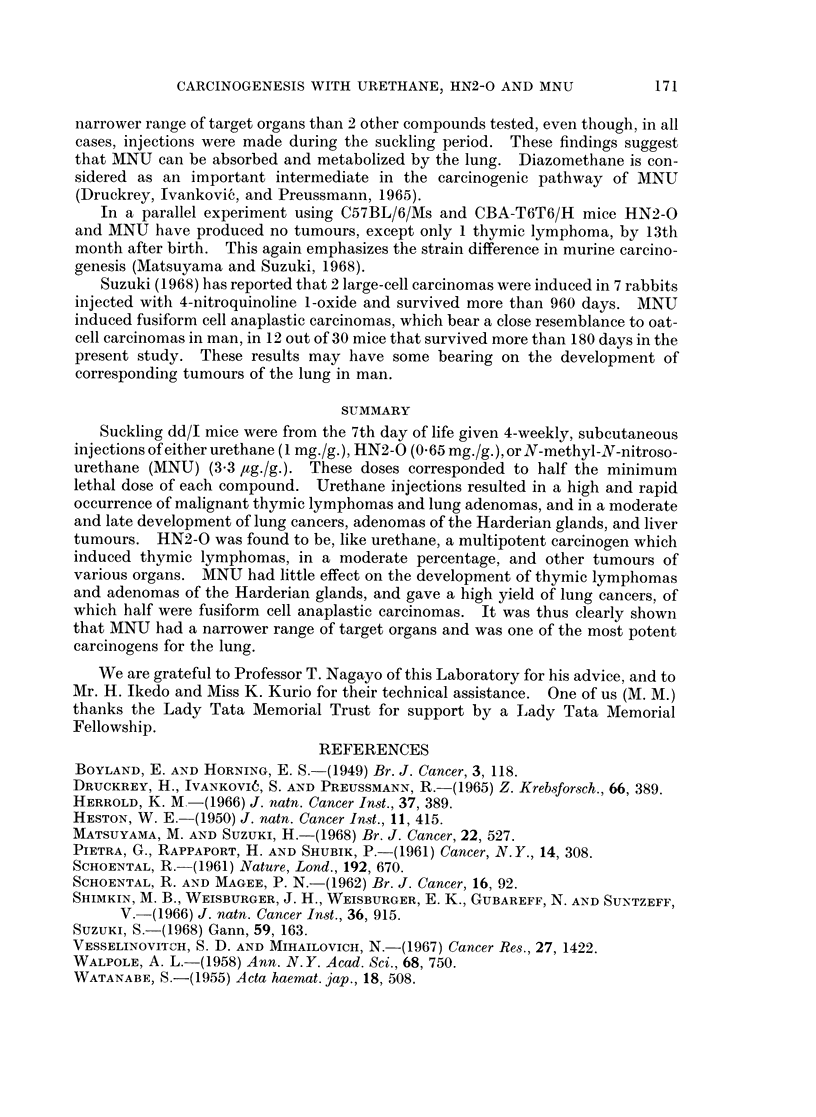# Carcinogenesis in dd-I mice injected during suckling period with urethane, nitrogen mustard N-oxide, and nitroso-urethane.

**DOI:** 10.1038/bjc.1969.24

**Published:** 1969-03

**Authors:** M. Matsuyama, H. Suzuki, T. Nakamura

## Abstract

**Images:**


### CARCINOGENESIS IN dd/I MICE INJECTED DURING SUCKLING PERIOD WITH URETHANE, NITROGEN MUSTARD N-OXIDE, AND NITROSO-URETHANE

M. MATSUYAMA, H. SUZUKI AND T. NAKAMURA*

*From the Aichi Cancer Center Research Institute, Nagoya, Japan*

Received for publication October 7, 1968

IT has been shown that lung cancers, including anaplastic carcinomas, could be induced in mice of the A/Jax, dd/I, and SMA strains when large amount of urethane (ethyl carbamate) was injected during the suckling period (Matsuyama and Suzuki, 1968). Lung cancers have also been produced in dd/I mice exposed to nitrogen mustard N-oxide, methyl bis-($\beta$-chloroethyl) amine-N-oxide hydrochloride (HN2-O), during the suckling period (Matsuyama and Nakamura, unpublished).

*N*-methyl-*N*-nitroso-urethane (MNU), which was reported as a carcinogen for the forestomach and oesophagus when administered orally (Schoental and Magee, 1962), releases diazomethane in alkaline solution and at neutral pH (Schoental, 1961), and inhalation of the latter compound by young rats induced lung adenomas and a squamous carcinoma of lung (Shoental and Magee, 1962). The present experiment was, therefore, undertaken to compare the carcinogenic effects, especially on the lung, of urethane, HN2-O, and MNU injected in suckling mice of the dd/I strain, which has been proved to be a moderately susceptible strain to urethane carcinogenesis in the lung (Matsuyama and Suzuki, 1968).

### MATERIALS AND METHODS

Female or male mice of the dd/I strain, originally obtained from Professor T. Ito of Hokkaido University, Sapporo, and bred in our laboratory by sister-to-brother mating since 1965, were used. Seven to ten litters each were given 4-weekly subcutaneous injections in the back with saline solution either of urethane, HN2-O, or MNU in amounts equal to half of the minimum lethal dose, starting at 7 days of age. A 10% solution of urethane (E. Merck, Darmstadt) was administered at a dose of 1 mg./g. of body weight. A 3% solution of HN2-O (Yoshitomi Pharmaceutical Ind., Osaka) was injected at a dose of 0·65 mg./g. MNU (K & K Lab. Inc., Plainview, N.Y.) was given at a dose of 3·3$\mu$g./g., as a 0·05% solution. As controls, 12 litters of mice were injected with saline alone at 0·01 ml./g., according to the same schedule. All the mice injected with urethane, HN2-O, MNU, or saline were kept separately from their mothers for about 3 hours to avoid maternal cannibalism. Litters were weaned at 5 weeks of age and separated according to sex. They were housed in aluminium cages with sawdust bedding and were given CMF diet (Oriental Yeast, Tokyo) and water *ad libitum*. The animals were allowed to survive their life span and were observed periodically for lymphomas and other tumours. When mice were found in poor physical condition they were killed and carefully autopsied. The lungs, Harderian glands, and other organs which showed grossly visible tumours were fixed in 10% formalin solution, sectioned and stained

* Present address: Department of Pathology, Nagoya City University Medical School, Nagoya, Japan.

with haematoxylin and eosin, by silver impregnation and by the periodic acid-Schiff reaction, if necessary, for microscopic examination.

### RESULTS

Deaths due to toxicity were rare, except in the case of mice injected with HN2-O in which 14% died during the injection period. However, delayed death due to reduced resistance against pneumonia was prominent in the mice injected with MNU and thus 10 mice (24%) were lost after weaning. Table I shows that the treatments during the suckling period in 3 groups significantly reduced survival days by induction of tumours of various types. There was no appreciable sex difference in survival days or tumour incidences and therefore the results for both sexes were pooled in each group (Table I).

In response to urethane or HN2-O, malignant thymic lymphomas developed in 70 and 57% of mice, respectively, and many of them died before the 180th day after birth which is the time needed for the development of lung cancer (Table I). A few of the MNU-treated mice developed thymic lymphomas, but lung cancer occurred in 26 out of the 30 mice that survived more than 180 days. Adenomas of the Harderian glands and liver tumours were also seen in the mice injected with urethane or HN2-O, whereas none of these tumours were observed in the MNU-injected mice (Table I). No tumour was found at the injected site in any group. In the mice injected with saline alone the only neoplasms seen were a few lung adenomas and non-thymic lymphomas.

Most of the lung adenomas were pearly-white, round masses, slightly raised and sharply delineated from the surrounding lung tissues, measuring 1–8 mm. in diameter. Histologically 2 types of adenomas could be differentiated; small circumscribed and large papillary adenomas. The former were located in the periphery of the lung and were closely packed columns of cells which were uniform, small, cuboidal or columnar in shape, with eosinophilic cytoplasm. The cellular columns were supported by a sparse stroma of mature fibrous tissue. The latter type of adenomas sometimes occupied the whole lobe with intrabronchial projections and showed more papillary structures. The cells of this type of adenomas were fairly large, cuboidal in shape and their cytoplasm were stained basophilic. Not rarely carcinomatous nodules, which had given rise to metastatic foci in the other organs, were seen in these papillary adenoma masses.

Lung cancers appeared as uneven surfaced nodules, 1–3 in number, measuring 4–16 mm. in diameter. The majority of cancers contained severe intrabronchial invasions and central necrotic masses, and accompanied severe thickening of the pleura and sometimes suprapleural cancerous deposits (Table II and Fig. 1). Some of the cancers had metastatic nodules in the other parts of the lungs, mediastinal tissues, chest wall, diaphragm, liver, and kidneys (Table II). Histologically 2 types of carcinomas, papillotubular adenocarcinoma and anaplastic carcinoma, were found (see Matsuyama and Suzuki, 1968). The former type of carcinoma consisted of large cuboidal cells, showing cytologic and structural atypism. They were associated with broader luminal spaces and interstitial connective tissue bundles (Fig. 2). Nuclei were vesicular and had from 1 to 3 prominent nucleoli. Cytoplasm stained basophilic. The latter type of carcinoma consisted of severely atypical, small, fusiform cells with numerous mitotic figures, showing high cellularity and destruction of the background lung structure. There was no apparent interstitium in them, except a few vessels (Fig. 3). They metastasized into more remote organs than did the papillotubular carcinomas (Fig. 4). In the lung and proximate metastatic foci, such as the mediastinal tissues, glandular structures were also intermingled with anaplastic masses, whilst the latter were predominant in the remote metastases. It is noteworthy that almost half of the lung cancers induced by MNU were anaplastic (Table II). No squamous cell carcinoma was found.

The histological characteristics of the neoplasms of the organs, such as the thymus, Harderian glands, and liver, in the mice were the same as previously described (Pietra, Rappaport and Shubik, 1961; Vesselinovitch and Mihailovich, 1967).

### DISCUSSION

The present results confirm those of the previous study (Matsuyama and Suzuki, 1968) in that urethane produced lung cancers, as well as multiple lung adenomas, in 52% of the animals of the dd/I strain that survived more than 180 days. Histological examination also revealed that the Harderian gland of the dd/I strain of mice has the same moderate susceptibility to the compound as the (C57BL $\times$ C3H)$F_1$ hybrid (Vesselinovitch and Mihailovich, 1967).

Injections of nitrogen mustard, methyl bis (2-chloroethyl)-amine hydrochloride, induced or accelerated lung adenomas in mice (Boyland and Horning, 1949; Heston, 1950; Shimkin *et al.*, 1966). Watanabe (1955) found that repeated intravenous injections of HN2-O in adult dd/N mice induced non-thymic lymphomas in 33% of the animals. The induction of lung cancers and malignant thymic lymphomas by 4-weekly injections of HN2-O at the higher tolerated doses, during the suckling period, is well illustrated in the present study. Walpole (1958) briefly described that skin painting of nonanoylethylenimine in random-bred mice of the Chester Beatty strain induced, in significant proportion, lymphosarcomas of thymic origin. Thus these results show the multicarcinogenicity of HN2-O. Extensive electron-microscopical examinations have resulted in a failure to detect virus-like particles in thymic lymphomas and lung tumours induced by injections of HN2-O or urethane (Matsuyama and Suzuki, unpublished).

Intragastric administration of MNU induced epidermoid carcinomas of the oesophagus and forestomach in rats (Schoental and Magee, 1962) and in Syrian hamsters (Herrold, 1966). Subcutaneous injections of this compound to adult mice and rats induced lung adenomas (Schoental and Magee, 1962). When injected during the suckling period MNU produced lung cancers, of which about half were anaplastic, in a high percentage of mice in the present experiment. This potent carcinogenicity for the lung is interesting since the carcinogen seems to have a

---

### EXPLANATION OF PLATES

FIG. 1.—Female mouse injected with MNU 4 times during the suckling period, age 384 days. Papillotubular adenocarcinoma contained central necrotic masses, and accompanied with severe thickening of the pleura and suprapleural carcinomatous deposit (upper left). H. and E. $\times$ 69.

FIG. 2.—Male mouse injected with HN2-O, aged 270 days. Papillotubular adenocarcinoma consisted of large cuboidal cells, in which some of them were necrotic and dark stained, with broader stromal bundles and luminal spaces. H. and E. $\times$ 379.

FIG. 3.—Female mouse injected with MNU, age 319 days. Fusiform cell anaplastic carcinoma with a few vessels. H. and E. $\times$ 436.

FIG. 4.—Same mouse shown in Fig. 3. Kidney metastasis of anaplastic lung carcinoma, including 3 necrobiotic glomeruli. H. and E. $\times$ 172.

Matsuyama, Suzuki and Nakamura.

Matsuyama, Suzuki and Nakamura.

narrower range of target organs than 2 other compounds tested, even though, in all cases, injections were made during the suckling period. These findings suggest that MNU can be absorbed and metabolized by the lung. Diazomethane is considered as an important intermediate in the carcinogenic pathway of MNU (Druckrey, Ivanković, and Preussmann, 1965).

In a parallel experiment using C57BL/6/Ms and CBA-T6T6/H mice HN2-O and MNU have produced no tumours, except only 1 thymic lymphoma, by 13th month after birth. This again emphasizes the strain difference in murine carcinogenesis (Matsuyama and Suzuki, 1968).

Suzuki (1968) has reported that 2 large-cell carcinomas were induced in 7 rabbits injected with 4-nitroquinoline 1-oxide and survived more than 960 days. MNU induced fusiform cell anaplastic carcinomas, which bear a close resemblance to oat-cell carcinomas in man, in 12 out of 30 mice that survived more than 180 days in the present study. These results may have some bearing on the development of corresponding tumours of the lung in man.

### SUMMARY

Suckling dd/I mice were from the 7th day of life given 4-weekly, subcutaneous injections of either urethane (1 mg./g.), HN2-O (0·65 mg./g.), or *N*-methyl-*N*-nitroso-urethane (MNU) (3·3 μg./g.). These doses corresponded to half the minimum lethal dose of each compound. Urethane injections resulted in a high and rapid occurrence of malignant thymic lymphomas and lung adenomas, and in a moderate and late development of lung cancers, adenomas of the Harderian glands, and liver tumours. HN2-O was found to be, like urethane, a multipotent carcinogen which induced thymic lymphomas, in a moderate percentage, and other tumours of various organs. MNU had little effect on the development of thymic lymphomas and adenomas of the Harderian glands, and gave a high yield of lung cancers, of which half were fusiform cell anaplastic carcinomas. It was thus clearly shown that MNU had a narrower range of target organs and was one of the most potent carcinogens for the lung.

We are grateful to Professor T. Nagayo of this Laboratory for his advice, and to Mr. H. Ikedo and Miss K. Kurio for their technical assistance. One of us (M. M.) thanks the Lady Tata Memorial Trust for support by a Lady Tata Memorial Fellowship.

### REFERENCES

Boyland, E. and Horning, E. S.—(1949) *Br. J. Cancer*, **3**, 118.
Druckrey, H., Ivanković, S. and Preussmann, R.—(1965) *Z. Krebsforsch.*, **66**, 389.
Herrold, K. M.—(1966) *J. natn. Cancer Inst.*, **37**, 389.
Heston, W. E.—(1950) *J. natn. Cancer Inst.*, **11**, 415.
Matsuyama, M. and Suzuki, H.—(1968) *Br. J. Cancer*, **22**, 527.
Pietra, G., Rappaport, H. and Shubik, P.—(1961) *Cancer, N.Y.*, **14**, 308.
Schoental, R.—(1961) *Nature, Lond.*, **192**, 670.
Schoental, R. and Magee, P. N.—(1962) *Br. J. Cancer*, **16**, 92.
Shimkin, M. B., Weisburger, J. H., Weisburger, E. K., Gubareff, N. and Suntzeff, V.—(1966) *J. natn. Cancer Inst.*, **36**, 915.
Suzuki, S.—(1968) Gann, **59**, 163.
Vesselinovitch, S. D. and Mihailovich, N.—(1967) *Cancer Res.*, **27**, 1422.
Walpole, A. L.—(1958) *Ann. N.Y. Acad. Sci.*, **68**, 750.
Watanabe, S.—(1955) *Acta haemat. jap.*, **18**, 508.